# Beyond Problem-Solving: Homeroom Teachers’ Reflective Practice as a Tool for Mental Health Support in Chinese Schools

**DOI:** 10.3390/bs15111510

**Published:** 2025-11-06

**Authors:** Huizhen Zheng, Qili Xie, Danyang Li, Guangrong Jiang

**Affiliations:** 1 Key Laboratory of Human Development and Mental Health of Hubei Province, School of Psychology, Central China Normal University, Wuhan 430079, China; psy_zhz@mails.ccnu.edu.cn; 2 School of Psychology, Central China Normal University, Wuhan 430079, China; 3 Department of Mental Health Education and Counseling Center, Guizhou Education University, Guiyang 550018, China; xieqilix@163.com; 4 Hubei Oriental Insight Mental Health Institute, Wuhan 430071, China

**Keywords:** student mental health, homeroom teacher, mental health education, multi-tiered systems of support, reflective practice, qualitative research, semi-structured interview, reflexive thematic analysis

## Abstract

This study explored the psychological characteristics of homeroom teachers’ reflective practice with a focus on student mental health, addressing a gap in empirical research. This study conducted semi-structured interviews with seventeen Chinese homeroom teachers and applied thematic analysis to examine how reflective practice supported mental health education. It also evaluated this practice from the perspective of multi-tiered systems of support (MTSS). The findings reveal the cognitive, emotional, motivational, and behavioral characteristics of homeroom teachers’ reflective practice. Cognitive characteristics centered on three aspects—the focus of reflection, the thinking process, and the formation or transformation of cognition—with student mental health being a primary concern. Emotional elements were less explicitly mentioned but were embedded in teachers’ narratives. Motivational characteristics comprised autonomy and physical–mental states, supporting or impeding reflection. Behaviorally, homeroom teachers engaged in silent, written, and dialogic forms of reflection, with silent reflection being common yet often undervalued. The study also indicated that homeroom teachers’ work in mental health education mainly involves MTSS Tier 1 and Tier 2, with insufficient collaboration with other professionals and characteristics distinct from traditional MTSS practices. Overall, the study highlights the multifaceted nature of reflective practice and its implications for enhancing school-based mental health education.

## 1. Introduction

Mental health issues among children and adolescents have become an increasingly prominent concern in China. Recent data show that over 16.9% of elementary and secondary school students^1^ are at risk of depression (Sun et al., 2025). In response to the increasing mental health needs, the Chinese government has issued policy guidelines that emphasize appointing full-time mental health educators in schools (Ministry of Education of the People’s Republic of China, 2021, 2023). In addition, China has been actively building an integrated student mental health system encompassing education, prevention, counseling, and intervention, and shifting its approach from a correction model to a prevention model. This transition highlights the need not only to strengthen the workforce of psychiatrists and full-time mental health educators, but also to build a broader support network including school administrators, homeroom teachers, parents, and peers (Fan et al., 2023; Zhao & Wang, 2021). This orientation also reflects the principles of multi-tiered systems of support (MTSS). MTSS is a population-based model of mental health prevention and intervention commonly used in schools, emphasizing proactive, comprehensive, and evidence-based support for students with varying level of needs. According to the MTSS framework, universal preventive measures should be used to promote students’ adaptive behaviors (i.e., Tier 1) and prevent problems from emerging, and targeted early interventions should be applied to less severe problems (i.e., Tier 2) in order to reduce the workload of intensive interventions for persistent and severe problems (i.e., Tier 3) (Arora et al., 2019; Qu et al., 2024).

In the comprehensive network of mental health support, homeroom teachers (Chinese: Banzhuren) play a key role (Yao et al., 2021), reflecting the strengths of China’s administrative class system and the homeroom teacher system. Chinese classes are often viewed as miniature communities, with members largely stable within the same class during a given educational stage and participating together in most academic and socialization activities (Li & Chen, 2013; Xiong & Sun, 2017). Each class is assigned a homeroom teacher, usually one of the main subject teachers. In addition to teaching, homeroom teachers are also responsible for class management, home–school communication, and mental health education (T. Wang & Yang, 2020). Compared with school mental health educators and general subject teachers, homeroom teachers have the closest daily contact with students, which enables them to develop the most in-depth understanding, notice psychological or behavioral difficulties more readily, and act as both the first responders and ongoing supporters in interventions (Yao et al., 2021; Yu & Zhang, 2023). One approach homeroom teachers use to implement mental health education is to integrate mental health education into daily class management by fostering positive teacher–student relationships and creating emotionally safe and inclusive class environments (Froiland et al., 2019; Jiang & Lin, 2000). Another approach is by providing targeted support to students experiencing psychological or behavioral difficulties (e.g., Liao et al., 2023; L. Wang, 2013). These two approaches mainly correspond to tiers 1 and 2 of the MTSS framework (Arora et al., 2019; Skaar et al., 2016). Empirical studies have fully demonstrated the positive role of homeroom teachers in shaping class climate and supporting students’ mental health (e.g., Han et al., 2004; Jiang, 2002; Xie et al., 2020). Given that a substantial proportion of elementary and secondary schools in China still lack full-time school mental health educators (Fu et al., 2024; Peng et al., 2021), the role of homeroom teachers in mental health education is especially prominent. However, enhancing homeroom teachers’ capacity to deliver mental health education remains a significant issue that requires further investigation.

Training in psychological knowledge and skills can somewhat improve homeroom teachers’ mental health education capacity (Pang, 2023; Wen, 2015), but such training alone often fails to address the complex challenges they face in practice (Amri, 2013; J. Wang et al., 2018). This paper argues that to effectively fulfill mental health education responsibilities, homeroom teachers require not only relevant knowledge and skills but also the capacity for reflective practice—critically examining their daily practice to gain new insights and improve future actions (Mohamed et al., 2022). The importance of homeroom teachers’ reflective practice lies not only in addressing ill-structured problems (Reed, 2016) but also in its impact on students’ mental health through teachers’ behavior, which can be positive or negative (Obsuth et al., 2017). Thus, the reflective practice emphasized in this study goes beyond immediate problem-solving and focuses on promoting students’ mental health.

Surveys indicate that most Chinese teachers reflect on their work (Tao, 2021; Q. Zhang, 2012). While non-homeroom teachers mainly focus on teaching reflection (e.g., Chang, 2023), the content of homeroom teachers’ reflection mirrors their diverse responsibilities (e.g., Liu & Zhou, 2020). Homeroom teachers’ reflective essays offer insight into their current reflective practice (Farrell, 2013). These essays suggest that such practice often encompasses components of mental health education. For example, some essays focus on students’ difficulties in school adjustment, interpersonal relationships, and emotional management. They also demonstrate homeroom teachers’ sensitivity to individual differences and a deliberate effort to adjust their approaches accordingly (e.g., Chen, 2023). Moreover, teachers’ accounts encompass both responsive interventions to unexpected incidents or individual students (e.g., Fang, 2023) and long-term initiatives aimed at improving the overall class environment (e.g., D. Wang, 2023). Lastly, homeroom teachers frequently evaluate the effectiveness of their actions based on whether students show positive changes (e.g., Ai et al., 2023). These indications suggest that homeroom teachers employ reflective practice in mental health education, and their work in this domain spans multiple tiers of MTSS.

However, the breadth and depth of homeroom teachers’ reflection still need improvement. In terms of breadth, published essays rarely addressed teachers’ reflection on their emotions and mental health. This might indicate underexplored areas in their reflection or may reflect the private nature of such topics, which reduces the likelihood of public sharing. In terms of depth, homeroom teachers’ attributions tended to be superficial. They seldom engaged in deeper self-inquiry, such as “Where does my impatience come from?” or “Why am I particularly reactive to this student?” Attributions that lack deep reflection tend to diminish the effectiveness of subsequent responses (Brun et al., 2021; Heffron et al., 2016). Empirical evidence has demonstrated that teachers’ personal factors, such as emotions, mental health, and behavioral motivations, exert a significant influence on students’ psychological development and academic achievement (Frenzel et al., 2021; Harding et al., 2019; Li & Ma, 2025). Therefore, it is necessary to include these aspects within the scope of teachers’ reflection.

In fact, the above discussion on breadth and depth also implies an important standpoint of this study—namely, viewing homeroom teachers’ reflective practice through the perspective of psychological counselors (see Aponte & Kissil, 2016; Ladany & Bradley, 2010). This standpoint is supported by the similarities between the two roles in terms of professional responsibilities (Chinese Psychological Society, 2018; McNaughton & Li, 2021), the interpersonal nature of their work processes (Pennings et al., 2018; Soma et al., 2020), and their experiences with countertransference (Cartwright et al., 2021; Sherry et al., 2021). The most significant similarity is their reliance on “the use of self” throughout the work (Morrissette, 2002). When teachers and counselors lack self-awareness of personal issues or interpersonal patterns, they may unintentionally harm those they work with. By contrast, through self-reflection, they can transform these personal traits into valuable resources in helping relationships (Aponte & Kissil, 2016). To improve the effectiveness of mental health education, teachers’ reflection should encompass the intrapersonal, interpersonal, and clinical dimensions (Chigwedere et al., 2021; DeCino et al., 2024).

Relying on homeroom teachers’ public accounts to understand their reflective practice has certain methodological limitations. For example, such public accounts often fail to authentically reflect teachers’ actions, emotions, and motivations (Hunter, 2021; Mann & Walsh, 2013). Additionally, educational narratives are fragmented and personal in nature, which makes it difficult to comprehensively or systematically capture the characteristics of homeroom teachers’ reflective practice. Unfortunately, empirical research remains scarce on homeroom teachers’ reflective practice, particularly in the context of mental health education. In contrast, studies on reflective practice among the broader teacher population have primarily focused on professional development and instructional improvement (McLeod, 2019; Pereira et al., 2023). These gaps constrain the value of reflective practice for mental health education and hinder the development of related intervention programs.

This study examines homeroom teachers’ reflective practice through the lens of student mental health education, focusing on their cognitive, emotional, motivational, and behavioral characteristics. It aims to provide empirical evidence to inform the enhancement of students’ mental health and teacher training. Specifically, this study investigates the key components of homeroom teachers’ reflective practice (e.g., focus, forms, thinking process, emotions, motivations, and influencing factors) and how reflective practice interacts with mental health education. Based on the findings, we offer recommendations for optimizing homeroom teachers’ reflective practice and homeroom teacher training to better support students’ mental health and analyze the unique role of Chinese homeroom teachers within MTSS. Furthermore, this study moves beyond reliance on publicly available reflective essays and obtains firsthand insights into homeroom teachers’ reflective practice through in-depth interviews.

## 2. Materials and Methods

### 2.1. Participants

The present study adopted the concept of information power proposed by Braun and Clarke (2022) to determine the sample size. Specifically, all interviews were conducted by the same interviewer and the interview phase was terminated when the collected data were assessed as sufficiently rich and relevant. Ultimately, the participants consisted of 17 homeroom teachers (13 women, 4 men) from various regions in China, all Chinese nationals. Participants ranged in age from 24 to 53 years (M = 35.4, SD = 9.4), with 0.3 to 33 years of teaching experience (M = 12.7, SD = 10.6) and 0.3 to 32 years as homeroom teachers (M = 10.3, SD = 10.4). All participants held at least a bachelor’s degree. Ten taught at the elementary level, five at the junior high level, and two at the senior high level.

### 2.2. Researchers

The data collection and analysis team consisted of four members, all Chinese and all trained in clinical and counseling psychology. The lead researcher, R1, a female doctoral student focused on student mental health and teacher professional development, was responsible for conducting interviews, transcribing data, and implementing the primary analysis. R2, a male PhD with the same research focus, contributed to discussions on the interview protocol and coding results. After the initial coding was completed, R3, a female PhD focused on counseling process-outcome and mental health literacy, served as the auditor of the coding results. In addition, R4, a clinical psychologist and senior professor with extensive practical and research experience in the field of homeroom teachers’ mental health education, provided ongoing guidance to R1 throughout the data collection and analysis process.

The research team has a strong background in Clinical and Counseling Psychology, which shaped the study’s focus on examining homeroom teachers’ reflective practices from a clinical and counseling perspective. This perspective allowed the study to move beyond traditional educational perspectives and identify gaps in homeroom teachers’ current reflective practice. However, it may have introduced selective attention and over-interpretation. To address such limitations, the team deliberately engaged with homeroom teachers during long-term research on mental health education in elementary and secondary schools, gaining insight into their actual work contexts. In this study, we continuously reflected on our professional background, positionality, and assumptions across all stages of the research, from study design to manuscript writing. We strove to ensure that interview questions aligned with teachers’ perceptions of their work and that data interpretations accurately represented the original data. In addition, an elementary homeroom teacher reviewed the coding results and generally endorsed them.

### 2.3. Data Collection

#### 2.3.1. Participant Recruitment

The study was approved by the Institutional Review Board (IRB) of the first author’s university in October 2022, prior to data collection. Homeroom teachers across China were recruited for the study. To be eligible, participants had to (a) serve as a homeroom teacher and (b) have relevant experiences or perspectives on “reflecting on the homeroom work” and be willing to share them. Participants were not required to engage in reflective practice regularly; rather, the study sought to include a diverse sample in order to comprehensively understand reflective practice among homeroom teachers.

The recruitment announcement for this study was delivered as a WeChat public article and was disseminated by individuals who had access to homeroom teachers. The article included a link to the registration form hosted on Wenjuanxing “https://www.wjx.cn/ (accessed on 3 November 2025)”, an online survey platform. Interested teachers could complete the form directly. The form included a multiple-choice item on whether they habitually reflect on their work. R1 contacted eligible respondents to provide detailed information about the study. Teachers who agreed to participate signed an informed consent form on Wenjuanwang “https://www.wenjuan.com/ (accessed on 3 November 2025)” and scheduled an interview. Teachers were sent the interview protocol in advance to facilitate reflection and recall.

#### 2.3.2. Interviews and Transcription

R1 drafted the interview protocol and sought revision suggestions from R2. Subsequently, R1 conducted pilot interviews with six homeroom teachers and refined the protocol based on the results, producing the final version for the formal interviews.

Recognizing that homeroom teachers’ mental health education may be embedded in their daily work, this study did not restrict the interviews to specific mental health activities. Instead, the interviews focused on teachers’ reflection on their everyday practice to understand how reflective practice is comprehensively applied in their work, including mental health education. The research purpose was translated into concrete interview questions that resonated with teachers’ lived experiences to elicit authentic and in-depth responses.

Two versions of the interview protocol were developed to accommodate participants with or without a habit of reflective practice: one for reflective practitioners (Version 1) and the other for non-reflective practitioners (Version 2). Table 1 compares the two versions. The full interview protocols are available in the Appendix A. Two participants were interviewed with the Non-reflective Practitioner protocol.

The semi-structured interviews were conducted by R1 via the Tencent Meeting platform (a widely used video conferencing service in China). With participants’ consent reaffirmed before each session, all interviews were audio-recorded. Upon completion, participants were thanked and received a small monetary reward via WeChat.

Two procedural measures were adopted to minimize participants’ tendency toward impression management and to support exploring personal experiences. First, individual interviews rather than focus group discussions were conducted to reduce the influence of others’ presence. Second, interviews were held in an undisturbed environment, with deliberate efforts to foster an atmosphere of trust, understanding, and openness. Based on the interviewer’s intuitive impressions during the sessions and an evaluation of data richness, participants demonstrated a high level of openness, resulting in relatively authentic interview data.

Seventeen formal interview recordings were collected and transcribed by R1, serving as the foundation for subsequent data analysis. The transcription process was conducted in two stages. First, initial transcripts were generated using an automatic speech recognition platform “https://www.feishu.cn/product/minutes (accessed on 3 November 2025)”. Second, each transcript was meticulously proofread by R1 against the original recordings. This study primarily adopted a denaturalized transcription approach (Point & Baruch, 2023), in which redundant or repetitive words were removed, sentence structures were refined, and punctuation was standardized to enhance readability and eliminate ambiguity, while preserving participants’ intended meanings. Participants’ verbal fillers and natural speech styles were retained. The researcher confirmed that these procedures did not alter the intended meaning, tone, or emphasis of the original narratives.

To uphold ethical standards in data handling, all audio files were renamed using numerical codes before uploading for transcription, and the resulting transcripts were labeled using the same naming system. In addition, any personally identifiable information (e.g., names, locations) within the transcripts was anonymized.

Excluding the openings and closings of the interviews, the total recording duration was 765 min, with individual interviews ranging from 27 to 81 min (M = 45.0, SD = 13.5). The transcripts totaled 174,000 Chinese characters, with individual transcripts ranging from 5244 to 19,249 Chinese characters (M = 10,220.7, SD = 3460.5).

### 2.4. Data Analysis

The aim of this study is to explore the psychological characteristics underlying homeroom teachers’ reflective practice and to identify core themes, rather than to pursue coding objectivity. Accordingly, the data were analyzed using Reflexive Thematic Analysis (Braun & Clarke, 2020). Reflexive Thematic Analysis conceptualizes researcher subjectivity as a resource for knowledge production rather than a must-be-contained threat to credibility. It also emphasizes an open and iterative coding process instead of relying on a predetermined coding framework. Moreover, Reflexive Thematic Analysis assesses coding quality through theoretical sensitivity, reflexivity, and interpretive depth, rather than through consensus between coders or inter-rater reliability (Braun & Clarke, 2021).

All interview data were processed with MAXQDA 2022, a qualitative data analysis software that supports thematic analysis (Uştuk, 2022).

The coding process in this study broadly followed Braun and Clarke’s (2021) six-step framework for Reflexive Thematic Analysis, with the inclusion of an auditor. The data analysis primarily employed an inductive, data-driven approach; however, the development of the four themes of cognitive, emotional, motivational, and behavioral characteristics was influenced by the research objectives, reflecting certain deductive, theory-driven elements. The analysis proceeded as follows:

1. During transcript proofreading, R1 revisited the data to familiarize themselves and form initial impressions of the interviews (first impressions were formed during the interviews).
2. R1 read all transcripts, drafted preliminary subthemes, and reflected on personal assumptions and biases. R1 recognized that her endorsement of reflective practice could bias her against participants who were less engaged in reflection. To address this, she consciously set aside her personal stance and treated low engagement in reflection as a phenomenon to explore. Reflection on assumptions and biases continued throughout the analysis.
3. R1 proceeded with the formal analysis, generating and iteratively refining codes until she developed a clear and systematic coding framework that fully captured the interview data. She regularly updated R4 on her progress and received guidance throughout the process.
4. R1 and R2 briefly discussed the coding, and R1 made corresponding revisions. R3 then conducted two rounds of audit and provided feedback. R1 and R2 discussed the feedback and finalized the results.

Drawing on Braun and Clarke’s (2022) description of good Reflexive Thematic Analysis, the study adopted the following measures to ensure credibility:

1. R1 continuously reflected on her own stance during the interviews and analysis stages, striving to maintain a neutral perspective.
2. R1, as the primary coder, had extensive experience in qualitative research.
3. R2, R3, and R4 provided feedback on the coding, enriching the analytical perspective.
4. R1 immersed herself in the data during analysis, striving to ensure that all codes faithfully represented the original data, and any revisions based on feedback were validated against the raw data.
5. The research team has long focused on mental health education in elementary and secondary schools and has extensive engagement with homeroom teachers, ensuring that data interpretation closely reflected teachers’ real-world context.
6. The coding results were reviewed by an elementary homeroom teacher, who largely endorsed them.

## 3. Results

Based on the research questions and interview data, four themes were identified, namely cognitive, emotional, motivational, and behavioral characteristics of homeroom teachers’ reflective practice. Theme 1, cognitive characteristics, consisted of the focus of reflection, the thinking process involved, and the formation and transformation of cognition. Theme 2, emotional characteristics, captured teachers’ emotions linked to reflective events. Theme 3, motivational characteristics, outlined factors that facilitate or hinder engagement in reflective practice. Theme 4, behavioral characteristics, presented common forms of reflection and the actions taken based on reflection. The thematic framework developed in this study is presented in Table 2. Themes and their categories are detailed below.

### 3.1. Cognitive Characteristics

Cognitive characteristics are categorized into three aspects: the focus of reflection, the thinking process, and the formation and transformation of cognition. Table 3 presents detailed characteristics.

#### 3.1.1. Focus of Reflection

The focus of reflection mentioned by homeroom teachers fell into seven categories, namely (1) Student Mental Health, (2) Class Management, (3) Parent-School Communication, (4) Teaching Routines, (5) Teacher–Student Relationships, (6) Unexpected Incidents, and (7) Teacher Emotion Management. The first six categories correspond to homeroom teachers’ professional responsibilities, while the seventh relates to their self-regulation.

The category **Student Mental Health** directly reflects homeroom teachers’ concern about students’ mental health. This category can be divided into five subcategories, namely specific students, attention to needs, harm reduction, student development, and mental health education. The most frequent subcategory (12 participants) was the attention given to students with emotional or behavioral problems, such as chronic low mood, fighting, and frequent absenteeism. The following frequent subcategories were “attention to needs” and “harm reduction” (each mentioned by four participants). For example, Participant 7 noted, “When handling class affairs, I need to consider students’ inner needs. Sometimes I put myself in their shoes to truly understand their feelings.” Participant 9 stated, “When addressing issues, I consider whether my approach might cause harm to students. Realizing that a lack of psychological understanding can lead to harm, I recently enrolled in a counselor training course.” The next subcategory, student development (mentioned by three participants), primarily involved maintaining student growth portfolios, encouraging goal setting, and facilitating students’ transition into the workforce. The use of formal thematic class meetings was mentioned by only two participants. These findings suggested that homeroom teachers primarily provide individualized support for students with specific issues and strive to promote positive interactions while minimizing negative ones. In contrast, using structured activities for mental health education remains rare among them.

Beyond Student Mental Health, other categories also revealed homeroom teachers’ concern for students’ mental health. In **Parent-School Communication**, teachers considered the potential impact on students when interacting with parents. Participant 10 recalled, “After we called the parent to school, he physically punished the child in front of us. It was conceivable that such behavior was even worse at home. Since then, we have been hesitant to call parents in, fearing further harm to the child.” In **Teacher–Student Relationships**, homeroom teachers reflected on the impact of positive teacher–student relationships on student development. In **Teacher Emotion Management**, teachers stressed the importance of managing their own emotions to prevent harm to students.

#### 3.1.2. Thinking Process

Participants’ descriptions of specific reflective events revealed a five-stage thinking process in their reflective practice. These stages consisted of (1) describing the problem or event, (2) evaluating the appropriateness of the behavior, (3) attributing causes, (4) planning and implementing future actions, and (5) evaluating outcomes.

**Description and Evaluation.** Descriptions of the problem or event typically included background, relevant individuals, and the event’s unfolding. This stage often occupied a substantial portion of the narrative and corresponds to the focus of reflection outlined above. Subsequently, teachers might evaluate the appropriateness of their behavior, drawing on criteria such as goal achievement, safety consequences, psychological impact on students, and professional ethics. For example, Participant 7 reflected, “I do not think I handled it well. I should have responded more gently, but I ended up saying something that hurt the student.”

**Attribution.** Subsequently, homeroom teachers typically engaged in causal attribution, which was mostly psychological in nature. Ranked by frequency, these attributions consisted of students’ psychological characteristics (n = 11), teacher-related factors (n = 10), parenting practices (n = 8), external environments (n = 6), and mental health risks (n = 1). Notably, all except “teacher-related factors” represented external attributions. From the perspective of Bronfenbrenner’s Ecological Systems Theory (Bluteau et al., 2017), most attributions involved students’ microsystems and chronosystems. Microsystem factors included parenting practices, teacher-related factors, and peer/internet influences—the latter being part of the external environments. Chronosystem factors referred to the sociohistorical context, which also belonged to the external environments.

Specifically, “students’ psychological characteristics” refer to their personality traits, psychological needs, and developmental stages. For example, Participant 10 noted, “Our rural school had many left-behind children living with grandparents. While their material needs are met, they often lack emotional care.” “Teacher-related factors” mainly concern their professional competence and emotional management abilities. For example, Participant 17 stated, “The result was not satisfactory because I acted too impulsively before thinking clearly about my purpose.”

“Parenting practices” primarily referred to students’ family parenting styles. Although participants did not explicitly use typological terms, they described parenting behaviors that closely aligned with parenting style theory (Darling & Steinberg, 1993). For instance, Participant 13 described an authoritarian style: “His father beats him severely when he misbehaves, sometimes even breaking the stick, so he is terrified of his father.” Participant 4 described a permissive style: “I found that her parents lack guidance in her daily habits and tend to spoil her.” Participant 1 described a neglectful style: “His parents work away from home and rarely engage in his upbringing. They know little about his interpersonal relationships.”

“External environment” referred to the influences of peers, the internet, and the broader sociohistorical context (e.g., the COVID-19 pandemic). Only one participant mentioned “mental health risks”, advising parents to seek psychiatric assessment to determine whether the student should continue attending school.

**Plan, Implement and Evaluation.** Based on their attributions, homeroom teachers often engaged in iterative cycles of “planning and implementing future actions” and “evaluating outcomes”. These actions reflected sustained attention to students’ mental health (see “Actions Based on Reflection”). Outcome evaluations typically focused on whether interventions had facilitated students’ habit formation, moral development, academic improvement, and mental health. For example, Participant 16 described helping a student with significant difficulties in family relationships, emotions, and academics. She consistently gathered information from the student and his parents to develop a conceptualization of the issues. Based on this, she repeatedly facilitated parent–child communication to improve their relationship. Outcome evaluations were conducted throughout the process. Eventually, the student rebuilt ties with his parents, grew more motivated, and was admitted to college.

#### 3.1.3. Formation or Transformation of Cognition

“Formation or transformation of cognition” described how teachers’ reflective thinking develops and evolves. This process was shaped by three factors, namely significant figures or events, psychological training, and work experience.

**Significant Events or Figures.** Significant events or figures often served as turning points that prompted participants to reflect more frequently or deeply on specific aspects of their work. Such events included job changes, witnessing educational injustice, and safety incidents. These positive or negative events profoundly altered participants’ cognition and prompted changes in their actions.

Significant figures can be summarized as “mentors”, including exemplary homeroom teachers from literature, teachers during one’s schooling, and colleagues or superiors encountered at work. Their impact on participants often stemmed not from direct instruction of reflective skills but from their demonstrating care for students, passion for work, and mastery of class management. Under the influence of such mentors, participants developed a stronger sense of educational mission and actively explored effective class management strategies, gradually exhibiting a state of “active reflection.”

**Psychological Training.** Of the 17 participants, 11 had psychological training. This included attending training programs (n = 7), obtaining relevant certifications (n = 6), joining regional psychological associations (n = 5), reading psychology books (n = 3), and completing undergraduate or graduate degrees in psychology (n = 2). Seven participants specifically described how such training influenced their reflective practice. It enabled them to interpret students’ behaviors considering developmental stages; to consider students’ motivations, emotions and needs; to prioritize active listening; and to apply positive discipline in daily interactions. A homeroom teacher with a psychology degree stated, “When students misbehave, I give them a chance to explain and try to understand their behavior from their perspective before deciding the next steps. This approach protects their self-esteem and shows genuine care” (Participant 1).

**Work Experience.** “Work experience” demonstrated a progression in reflective practice at different career stages. Early in their roles, homeroom teachers focused on task completion with minimal reflection. As experience grew, they developed personal insights into class management and became more reflective. Participant 5 shared, “In my first two or three years, I just followed the school’s arrangements. With more experience, I began forming my views and thinking more actively about class development.”

### 3.2. Emotional Characteristics

Emotional characteristics were reflected in emotions related to the reflective events. Without deliberate prompting, homeroom teachers infrequently expressed emotions when describing impressive reflective events. Of the 17 participants, nine did not mention any emotions, and five of the remaining eight offered only brief descriptions, such as “I was terrified and did not know what to do” (Participant 7). Among the other three, Participant 13 reported deliberate emotional awareness: “When something bad happens, I try to identify how I feel. It has become a habit.” Participant 14 recognized how personal history shaped emotional responses: “Even a serious look from my father made me afraid. Growing up like that, I became strict with my students.” Participant 15 recognized how motivation influenced emotional responses: “To be honest, I was so angry at the student because I feared being blamed by my supervisor, who had repeatedly emphasized that students must keep their exam admission tickets safe.”

Notably, homeroom teachers seemed to express their emotions indirectly through narratives. For example, one participant shared multiple cases during the interview, subtly conveying emotional undertones. Some excerpts are presented below; to maintain brevity, certain parts are summarized:

“All homeroom teachers say that this graduating cohort is difficult to manage. When I was teaching them in Grades 7 and 8, no one in my class ever took leave. But after the class was reshuffled in Grade 9, the problem of frequent absences, which had been observed in other classes, began to appear in my class as well. One student, in particular, was very disengaged. He seemed weary whenever he was in the classroom. To address this issue, his parents came back from another city …[description of communication with the student’s parents]… Last night during self-study session, I had him copy the content of an online course in my office …[description of observing the student during this process]… I really wish the school had an institution to manage such disengaged students; otherwise, they might affect others.”

“Students in my class said I was the best teacher they had ever had. I think this may be because I was able to understand them. The family education training I attended emphasized understanding students’ inner needs and respecting their perspectives. Perhaps that is how I could connect with them effectively. …[Description of a specific incident regarding a student]… Another strength of mine is that I praise students for even the smallest achievements. For example, when I taught them how to arrange trash bins and chalk, I observed their performance and offered praise whenever they did it correctly.”

“Last year, I successfully obtained a mental health instructor certificate, which inspired many others to pursue it as well. My colleagues often comment on my eagerness to learn. I explain that I did not realize the importance of learning when I was young, so now I strive to expand my knowledge. I am confident that my commitment to learning can inspire others.”

From the excerpts above, we can discern the participant’s rich emotional landscape, including concern for students’ academic development, anxiety over students’ reduced school engagement, and pride in receiving recognition from students and colleagues. However, this participant did not explicitly express these emotions during the interview.

### 3.3. Motivational Characteristics

Motivational characteristics described factors that facilitated or hindered engagement in reflective practice. These characteristics were categorized into Autonomy and Physical-Mental State. Specific characteristics are presented in Table 4.

#### 3.3.1. Autonomy

**Intrinsic Motivation.** Intrinsic motivation was divided into five subcategories, namely (A) The Need for Problem-Solving, (B) Perceived Benefits, (C) A Sense of Identification, (D) Growth Mindset, and (E) The Need for Career Development.

**(A) The Need for Problem-Solving.** The presence of unresolved problems often triggers teachers’ reflective practice. Participant 7 stated, “To ensure task quality while also easing my workload, I began to reflect and to make some adjustments.” Some participants noted that the frequency of their reflection varied with the complexity of the issues. For example, Participant 8 stated, “When issues get tough, I reflect more; when things are under control, I reflect less.”

**(B) Perceived Benefits.** Homeroom teachers engage in reflective practice primarily due to its tangible benefits. Reflective practice positively impacts homeroom teachers’ work performance, mental well-being, and personal reputation, and achievement.

Benefits to work performance consisted of three main aspects: (a) Enhancing management strategies and outcomes, as Participant 1 noted, “Reflection helps me anticipate students’ adjustment challenges and offer early psychological support, which in turn enhances their coping capacities”; (b) Fostering greater respect for students, particularly regarding their self-esteem, autonomy, and individual differences, as participant 12 stated, “Through reflection, I began speaking more gently and humorously with students, which helped protect their self-esteem”; (c) Improving orderliness and efficiency, as Participant 11 explained, “Homeroom duties are demanding, but through engaging in reflection, I identified effective strategies that make the work more efficient.”

Benefits to mental well-being consisted of reducing stress, increasing acceptance, and enhancing the sense of fulfillment. Participant 4 stated, “I experienced irritability but gradually accepted the situation through self-adjustment.”

Benefits to personal reputation consisted of recognition from students, parents, and colleagues. Participant 4 said, “My colleagues found my strategies were always effective and called me Golden Ideas.” Benefits to personal achievement were demonstrated by honors like “Outstanding Homeroom Teacher” or awards in competitions. As Participant 11 stated, “Reflection helped me compile practical cases for publication and competitions, which brought me tangible benefits.”

**(C) A Sense of Identification.** A sense of identification was manifested by participants’ appreciation of both their work and the value of reflective practice. This recognition facilitates engagement in reflective practice, whereas a lack of it hinders such engagement. One participant who actively engages in reflection said, “I want my students and class to be better, so I am always seeking ways to solve problems” (Participant 6). In contrast, a participant who rarely engaged in reflection expressed resistance and frustration, “Being a homeroom teacher feels like a forced task. The school provided no training, and I feel pushed onto the stage unprepared” (Participant 9).

**(D) Growth Mindset.** Growth mindset was characterized by homeroom teachers’ positive expectations for capacity development, situation improvement, and proactive problem-solving orientations. Several participants expressed such beliefs. For example, Participant 7 noted, “It is okay to perform poorly at first; as long as I continue learning, progress will follow.” Participant 6 shared, “I have already learned from the experience. Dwelling on self-blame would only disrupt my daily work, so I choose to move on.” These participants were found to be actively engaged in reflective practice.

**(E) The Need for Career Development.** The need for career development motivated teachers to engage in reflective practice by linking it to their long-term professional goals. Participant 15 stated, “I hope to advance in my career, such as becoming a teaching expert. From this perspective, reflection is essential.”

**Extrinsic Motivation.** Externally imposed requirements tended to undermine participants’ initiative to engage in genuine reflection, sometimes reducing the reflection to a performative task aimed at pleasing superiors. Participant 17 shared, “Writing the homeroom teacher report feels like handing in homework, which makes me somewhat reluctant. Since our superior prefers impressive-looking reports, I often adapt online templates to suit his preferences. However, when I reflect for myself, I focus more on real challenges.” Participant 2 compared personal journaling with work reflections: “Journaling was relaxing. I write whatever and however much I want. But writing work reflections feels like an added task, as if I am working overtime.”

#### 3.3.2. Physical-Mental State

Several participants noted that poor physical-mental states (e.g., limited time and energy, high work pressure, and unfavorable psychological conditions) reduced the frequency and depth of reflection. Participant 11 stated, “When I am dealing with daily trivialities or experiencing negative emotional states, I prioritize handling these matters and do not reflect on my work.” Participant 3 stated, “I can reflect on problems that need immediate attention. However, I am less willing to think about issues like how to motivate students because of emotional fluctuations and being preoccupied with personal matters”.

### 3.4. Behavioral Characteristics

Behavioral characteristics were manifested in two key aspects, namely the common forms of reflection and the actions based on reflection. Specific characteristics are presented in Table 5.

#### 3.4.1. Forms of Reflection

Forms of reflection were classified into three categories, namely (1) Silent Reflection, (2) Written Reflection, and (3) Dialogic Reflection.

**Silent Reflection** was mainly an individual process and can be categorized into three types, namely reflection triggered by events, reading, and training. Among these, event-triggered reflection corresponds to reflection-in-action (Currano & Steinert, 2012), while reading- and training-triggered reflection demonstrated how knowledge input stimulates reflective thinking. Notably, some participants initially did not recognize silent reflection as a legitimate form of reflection and even classified themselves as “non-reflective practitioners” due to the absence of written output. This perception shifted during interviews. Participant 1 explained, “Before the interview, I knew I sometimes engaged in thinking but did not realize this counted as reflection. I had always assumed that reflection required writing it down.” Upon realizing that silent reflection qualifies as reflection, Participant 17 remarked, “Every homeroom teacher is constantly reflecting, and such ongoing reflection, to varying degrees, contributes to our professional growth.”

**Written Reflection** emphasized the use of text as a medium for reflection. While the writing process is typically carried out individually, the reflective artifacts may serve a communicative function. As Participant 14 described, “I wrote down cases related to class management and posted them on WeChat Moments. Many people read them, gave me likes, or left comments.” Written reflections were categorized into daily textual records and semester or annual reports. The former often stems from personal habits of reflection and documentation, whereas the latter is usually mandated by school administrators. Some participants reported challenges in maintaining routine records. Participant 4 explained, “I did want to document my everyday reflections, but I could never keep it up. One reason was that my workload was overwhelming, which made it difficult to find the time. Another reason was that I had not developed the habit, like setting aside dedicated time for it.”

**Dialogic Reflection** emphasized verbal communication as a medium for reflection, namely “informal exchanges” and “organized discussion meetings”. Informal exchanges referred to spontaneous conversations between homeroom teachers and their colleagues during daily work or when encountering specific challenges. Such informal exchanges were considered a crucial means of professional development. Participant 1 shared, “Regular discussions in the office facilitate the learning and professional development of novice teachers like me.” Discussion meetings are typically organized by education authorities or schools. In addition to addressing unresolved issues, these meetings also provide opportunities to share successful practices. This form of reflection also played a facilitating role. Participant 11 noted, “The discussion groups organized by the district education bureau prompt us to reflect daily on our work, which has helped me develop this habit.”

#### 3.4.2. Actions Based on Reflection

Reflections often guided subsequent actions, vividly illustrating the concept of “reflective practice.” As noted earlier, homeroom teachers frequently focus their reflections on student mental health, and their subsequent actions continue this focus. For instance, Participant 4 addressed a student’s behavioral issues through story discussions in class meetings while safeguarding the student’s self-esteem. In other cases, reflection prompted broader changes in class management style. Participant 15 harshly reprimanded a student and later regretted the psychological harm he caused. After reflection, he recognized the need to improve emotional management and consciously adopted a calmer class management style.

## 4. Discussion

Previous studies have primarily focused on the role of reflective practice in promoting teacher professional development and improving teaching quality (McLeod, 2019; Saadet & Yusuf, 2021). This study expands the scope by examining the application of reflective practice in the work of homeroom teachers. Specifically, it systematically examines Chinese homeroom teachers’ cognitive, emotional, motivational, and behavioral characteristics during reflective practice, emphasizing aspects related to student mental health. This research fills a gap in empirical studies on homeroom teachers’ reflection on student mental health.

### 4.1. Homeroom Teachers’ Mental Health Education Role Under the MTSS Framework

The most significant finding of this study is that homeroom teachers regarded students’ mental health as a central focus of reflection and decision-making. Notably, the interview protocol focused on homeroom teachers’ reflection on daily work and did not explicitly mention mental health. Thus, the spontaneous emergence of mental health-related content highlights teachers’ deliberate commitment to mental health education. This aligns with the findings of Yao et al. (2021) and supports the emerging model of “education as mental health therapy” in school mental health education (Cruz et al., 2021). Homeroom teachers’ mental health practice can be interpreted from multiple perspectives within the MTSS framework.

#### 4.1.1. Homeroom Teachers’ Extensive Involvement in the MTSS Process

Since the primary aim of this study was not to investigate homeroom teachers’ roles in mental health education within the MTSS framework, the interview questions did not address their collaboration with other mental health professionals. Based on the interview data, homeroom teachers’ involvement in mental health education primarily corresponds to Tier 1 and Tier 2 of MTSS, while also involving follow-up and support for Tier 3 activities. Their role spans the entire process of mental health education under the MTSS framework (Qu et al., 2024).

The cases of student absenteeism provided by two participants in this study aptly illustrate homeroom teachers’ extensive involvement. In the MTSS framework, school absence serves as an early warning indicator of student difficulties, but its impact must be interpreted alongside other student data (Kearney et al., 2025). In these cases, homeroom teachers evaluated the impact of absences by integrating additional student information and inferred that absenteeism was associated with school adjustment and academic challenges. Teachers then enhanced collaboration with parents and intervened through multiple channels, including monitoring student attendance, providing support for school adjustment, offering guidance on parent–child relationships, and advising parents to seek professional support from mental health clinics. Their involvement spanned the full spectrum of MTSS, from Tier 1 early prevention to Tier 3 intensive intervention (Kearney et al., 2025).

Homeroom teachers’ extensive involvement is largely related to the comprehensive influence of the class system and homeroom teacher system on students’ academic and daily lives in Chinese elementary and secondary schools, as well as the multifaceted responsibilities of homeroom teachers (Li̇ & Chen, 2013; Ministry of Education of the People’s Republic of China, 2006). Given the importance of homeroom teachers in mental health education and their limited professional expertise (Shang et al., 2025), it is necessary to provide them with more systematic and in-depth theoretical and practical training in mental health education to enhance their corresponding competencies.

#### 4.1.2. Fragmentation in School-Based Mental Health Systems

In this study, no participant reported referring students to or working with school mental health educators, which reflects the fragmentation of teaming and information sharing within comprehensive school-based mental health systems (Cowell, 2019). This fragmentation may lead to homeroom teachers being overburdened and lacking necessary professional support when dealing with complex cases (Shang et al., 2025). Previous research has also found that teachers rarely recommend professional help, speculating that stigma associated with mental illness and lack of training are contributing factors (Yao et al., 2021). This study partially agrees with these interpretations and further proposes that the limited referrals may also be related to a shortage of full-time mental health professionals in Chinese schools (Zhu, 2025). This further highlights the importance of homeroom teachers in the current MTSS framework of school mental health education. To provide timely and effective support to students in need, China’s education system still needs to strengthen the professional workforce for mental health education. It must also establish a more integrated prevention and intervention system that involves hospitals, communities, school administrators, mental health educators, homeroom teachers, and families. Finally, the system should enhance collaboration among professionals (Ministry of Education of the People’s Republic of China, 2023).

#### 4.1.3. Homeroom Teachers’ Mental Health Education Versus Conventional MTSS Models

Homeroom teachers’ mental health education reflects the principles of MTSS while exhibiting characteristics distinct from conventional MTSS models. First, while MTSS typically uses structured programs, such as formal curricula, group counseling, and individualized education plans (Kearney et al., 2025), Chinese homeroom teachers embed mental health education into their daily classroom management and teacher-student interactions, which is less formalized. Second, while MTSS emphasizes evidence-based interventions (August et al., 2018), the strategies taken by homeroom teachers are often based on experience and flexible application of theory. Finally, while MTSS relies on systematic student assessments to guide interventions (Clopton et al., 2024), homeroom teachers in this study rarely conduct such assessments and instead engage in a “practice-reflection” cycle based on long-term knowledge and real-time tracking of specific students. Homeroom teachers’ practice of embedding mental health education within daily classroom management and teacher-student interactions may facilitate ongoing mental health support, decrease stigma, and promote student adaptation (Mackenzie & Williams, 2018). It also lowers the financial and human resource costs of mental health education, making it feasible even in resource-limited regions and schools (Kearney et al., 2025). This study highlights the advantages of how Chinese homeroom teachers provide mental health education through the reflective practice of their daily work. Future research could incorporate this approach into the MTSS framework to broaden our understanding of mental health education practices in China and enrich MTSS implementation.

### 4.2. Emotions and Motivations Under-Recognized

Another notable finding is that homeroom teachers tended to overlook their emotions and motivations. In interviews, they rarely mentioned emotions or motivations related to reflective events, and even less frequently treated them as objects of reflection. Previous studies also found that reflectors focus more on cognition and behavior than on emotion. For instance, analyses of students’ reflective writing showed a preference for behavioral descriptions and logical analyses, with little emotional expression (Jung et al., 2021; Lin et al., 2016). In addition, we found an emphasis on behavioral motivations only within the domain of counselors’ reflective practice (Chigwedere et al., 2021; DeCino et al., 2024; Ladany & Bradley, 2010).

However, this study also found that teachers’ emotions and motivations might be implicitly embedded in their narratives. In the interviews, teachers’ accounts of reflective practice largely focused on detailed descriptions of problems or events. A large body of educational narrative, as well as findings by Catalana (2020) and Kremzer (2023), also suggests that teacher reflection is characterized by high narrativity. One possible explanation is that narrative serves emotional and motivational functions. Through storytelling, teachers may express their emotions and fulfill specific interpersonal motives, such as seeking socially desirable evaluations (Pasupathi et al., 2017; Veglia & Fini, 2017). The quotation from one participant in Section 3.2 illustrates the emotional and motivational elements embedded in homeroom teachers’ reflection, although these elements often remain at a subconscious level and rarely become the focus of further reflection. Based on the analogy drawn in Section 1 between homeroom teachers and counselors, we recommend that teachers attend to their emotions and motivations and consider how these elements affect their students. This may help deepen their understanding of reflective events and support more fundamental changes in practice (DeCino et al., 2024; Heffron et al., 2016).

From the perspective of teacher training, differences in the content of reflections between teachers and counselors can be traced to disparities in their respective training systems. Teachers’ reflective frameworks tend to focus on analyzing and improving actions, with case discussions focusing on students and specific action strategies (Moussa-Inaty, 2015; Thompson, 2021). In contrast, counselors’ frameworks go beyond these aspects to emphasize awareness and reflection on personal factors, a focus embedded throughout counselor training and supervision (Aponte & Kissil, 2016; Morrissette, 2002). To enhance the impact of reflection on homeroom teachers’ practice, it is necessary to draw on counselors’ reflective-practice training system. This can be achieved through two approaches: (1) inviting professionals with Clinical and Counseling Psychology backgrounds and facilitation skills to serve as facilitators in homeroom teacher training, and (2) providing existing facilitators with professional training that draws on counselors’ reflective practice, with the aim of expanding their facilitation skills.

### 4.3. Factors That Facilitate or Hinder Reflective Practice

#### 4.3.1. Psychological Training

This study also found that psychological training facilitates homeroom teachers’ reflective practice. Most participants had some form of psychological training, which prompted them to pay greater attention to students’ psychological needs and to adopt psychologically supportive approaches. This finding aligns with Qin et al. (2025) and Reeve et al. (2020). Prior studies have also found that limited mental health training impedes teachers from effectively delivering mental health education (Dinnen et al., 2024; Yao et al., 2021). In recent years, Chinese educational authorities have strengthened psychological training for pre-service and in-service teachers to improve the overall educational environment and support students’ mental health (Pang, 2023; X. Zhang, 2024). This study and related findings offer strong empirical support for the practical value of these policy measures. Chinese educational authorities and school administrators could actively promote psychological training programs for homeroom teachers. These programs would help teachers acquire more professional and systematic psychological knowledge and skills and enhance the effectiveness of their reflective practice.

Homeroom teachers demonstrated a pragmatic orientation towards learning psychology, aligning with characteristics of adult learning (Machynska & Boiko, 2020). When attributing student behaviors to parenting practices, they described three suboptimal parenting styles in colloquial terms and recognized the impact of family dynamics on school education. Throughout the interviews, although homeroom teachers frequently conveyed psychological principles and expressed concerns for students’ mental health, they rarely made explicit reference to specific psychological theories. This underscores the importance of integrating psychological theories with the practical work of homeroom teachers when disseminating psychological knowledge to them (Horn et al., 2024).

#### 4.3.2. Motivational Factors

Prior research has predominantly conceptualized reflective practice as a skill, with little exploration of how it may be influenced by individual motivational factors (e.g., Kiemer et al., 2015; Mohajer et al., 2024). Through qualitative analysis, the current study reveals that motivation plays a critical role in influencing teachers’ reflective practice. The theme of “motivational characteristics” identifies two types of factors that influence teachers’ motivation to engage in reflective practice, namely autonomy and physical-mental state. Autonomy, one of the three basic psychological needs in Self-Determination Theory (SDT), refers to perceiving one’s actions as self-directed, thereby enhancing engagement (Ryan & Deci, 2000). Drawing on SDT, this study classified some motivational factors as either intrinsic or extrinsic based on perceived autonomy. The findings suggest that intrinsic motivations tend to promote active and deep reflection, whereas extrinsic motivations that do not align with teachers’ genuine needs may diminish their reflective engagement and even provoke resistance. Prior research has similarly warned that reflection can become mechanical or superficial when disconnected from authentic personal development (Ramsey, 2010). Therefore, school administrators and homeroom teacher training providers should actively foster teachers’ intrinsic motivation, and minimize the potential hindrance caused by external demands.

Beyond autonomy, the study also found that a poor physical-mental state can weaken the willingness to engage in deep reflection. More concerning, prior research has shown that teachers’ negative emotional states may trigger harmful behaviors toward students (Ali et al., 2019). This suggests a dual detrimental impact: teachers who most need reflective practice to mitigate negative impacts on students are often the least able to engage in it (Atashpanjeh et al., 2020). Given that homeroom teachers experience greater emotional exhaustion than their non-homeroom colleagues (Li et al., 2022), administrators and training providers should pay close attention to homeroom teachers’ overall well-being. This involves fostering a supportive organizational climate and providing resources for physical-mental regulation (McLean et al., 2017; Zang & Jiang, 2023).

### 4.4. Silent Reflection That Is Undervalued

This study categorized homeroom teachers’ reflection into silent, written, and dialogic reflection forms. Previous research has focused mainly on the latter two forms, possibly as they are more observable and easier to implement in interventions (e.g., Firestone et al., 2020; Mena-Marcos et al., 2013). In contrast, due to its implicit nature, silent reflection tends to be overlooked by practitioners and researchers. This is echoed by some participants who expressed dissatisfaction with their lack of written reflection. Similarly, prior researchers have suggested that “thinking only” represents a lower level of reflection than writing reflective journals or engaging in discussions (e.g., M. Wang, 2015). However, no clear evidence suggests silent reflection is less effective than the other two forms. Moreover, silent reflection often serves as the only viable option when homeroom teachers must make quick decisions in practice (Manen, 1991). From a practical standpoint, enhancing the quality of silent reflection is a key goal of teacher reflection. Therefore, it is necessary to place greater emphasis on silent reflection and to improve its quality indirectly through written and dialogic reflection (Cattaneo & Motta, 2021). To achieve this, future training on homeroom teachers’ reflection should clarify the prevalence and legitimacy of silent reflection, while also guiding teachers to consciously transfer the cognitive frameworks of written and dialogic reflection into silent reflection.

### 4.5. Limitations and Future Directions

This study systematically examined the psychological characteristics of homeroom teachers’ reflective practice. It also provided insights into the value of this practice for student mental health education. However, the study’s design and implementation had several limitations that warrant further improvement and investigation.

First, the sample’s representativeness is somewhat limited. Although participants were recruited nationwide from different educational stages to ensure some diversity, purposive sampling inherently involves a risk of selection bias (Newington & Metcalfe, 2014; Stratton, 2024). Specifically, due to the recruitment criteria and the psychological background of the research team disclosed in the recruitment materials, the study may have disproportionately attracted individuals with a higher tendency for reflection and an interest or background in psychology, which could affect the generalizability of the findings (Palinkas et al., 2015). Future research should adopt stratified purposive sampling to include homeroom teachers with varying reflection tendencies and training backgrounds, while minimizing the salience of the research team’s professional identity in recruitment. These adjustments would allow for a richer understanding of the diversity in homeroom teachers’ reflective practice and enhance the comprehensiveness and credibility of the conclusions.

In addition, the study relied on a single data source. The findings were based solely on homeroom teachers’ narratives, without input from students, parents, or colleagues. Additionally, the study did not include cross-validation of teachers’ actual behaviors. These gaps may prevent a complete understanding of how reflective practice affects mental health education. Future research should draw on multiple sources, such as student feedback, peer evaluations, parent-school communication records, and classroom observations, to establish a cross-validation system and strengthen the credibility of the findings (Wei et al., 2023; Williams & Hebert, 2020).

Furthermore, emotion-related content in the interviews was limited. Although emotions are critical in initiating, sustaining, and deepening reflection (Eikey et al., 2021), participants rarely mentioned their emotional experiences. This made it difficult to examine how emotions interact with reflective practice. The lack of emotional content may be related to the interview prompts, personal communication styles, or cultural norms about emotional expression (Immordino-Yang et al., 2016; Siouti & Ruokonen-Engler, 2025). Future research should employ emotion-eliciting interviews or the emotional recall technique (Klemfuss & Musser, 2020; Peplak & Klemfuss, 2022) to help homeroom teachers better identify and articulate their emotions associated with reflective events. Moreover, homeroom teachers’ retrospective accounts in the interviews may not accurately represent their reflective processes at the time of the events, especially when a significant amount of time has passed (Mason et al., 2024; Ottenstein & Lischetzke, 2020). Therefore, future research should include contemporaneous reflective journals to capture a more authentic reflection process.

Finally, this study did not directly address MTSS in its design. In fact, it was part of the “Comprehensive Study on Reflective Practice of Chinese Elementary and Secondary School Homeroom Teachers,” with the aim of gathering baseline data on the current state of homeroom teachers’ reflective practices for future intervention programs. Therefore, the interview questions did not specifically address MTSS. Future research should involve school administrators, mental health educators, and homeroom teachers to deepen our understanding of the mental health service system in Chinese elementary and secondary schools.

## 5. Conclusions

Chinese homeroom teachers actively take responsibility for students’ mental health education and participate in various tiers of the Multi-Tiered Systems of Support. As part of this role, they often treat student mental health as a key focus of reflection and decision-making. However, teachers had limited awareness of their own emotions and motivations. In addition, homeroom teachers had limited collaboration with other professionals in the school’s mental health support system, with most of the work being conducted within the class. Furthermore, teachers with psychological training demonstrated greater sensitivity and professionalism in their reflective practice. The findings also suggested that teacher autonomy and physical-mental states could either facilitate or hinder their reflective practice. Finally, teachers employed various forms of reflection; silent reflection was common yet often undervalued.

Future training in psychology and reflective practice for homeroom teachers should be tailored to these realities. More importantly, the Chinese education system needs to strengthen the professional workforce in mental health education and establish a more integrated prevention and intervention system.

## Figures and Tables

**Table 1:** Comparison of the Two Versions of the Interview Protocols.

	Version 1 (Reflective Practitioner)	Version 2 (Non-Reflective Practitioner)
Section 1	Basic Information about “Reflecting on Homeroom Work Relevant Experiences Common Forms Significant Figures or Events	Basic Information about Challenges in Homeroom Work Presence of Challenges Strategies for Addressing Challenges
Section 2	Perspectives on “Reflecting on Homeroom Work Benefits Challenges Influencing factors	Perspectives on the Role of “Reflecting on Homeroom Work”

**Table 2:** Thematic Framework.

Themes	Categories
Cognitive Characteristics	Focus of Reflection Thinking Process Formation or Transformation of Cognition
Emotional Characteristics	
Motivational Characteristics	Autonomy Physical-mental State
Behavioral Characteristics	Forms of Reflection Actions Based on Reflection

**Table 3:** Cognitive Characteristics of Homeroom Teachers’ Reflective Practice.

Categories	Subcategories
Focus of Reflection	(1) Student Mental Health (2) Class Management (3) Parent-School Communication (4) Teaching Routines (5) Teacher–Student Relationships (6) Unexpected Incidents (7) Teacher Emotion Management
Thinking Process	(1) Describing the Problem or Event (2) Evaluating the Appropriateness of the Behavior (3) Attributing Causes (4) Planning and Implementing Future Actions (5) Evaluating Outcomes
Formation or Transformation of Cognition	(1) Significant Events or Figures (2) Psychological Training (3) Work Experience

**Table 4:** Motivational Characteristics of Homeroom Teachers’ Reflective Practice.

Categories	Subcategories
Autonomy	(1) Intrinsic Motivation (2) Extrinsic Motivation
Physical-Mental State	

**Table 5:** Behavioral Characteristics of Homeroom Teachers’ Reflective Practice.

Categories	Subcategories
Forms of Reflection	(1) Silent Reflection (2) Written Reflection (3) Dialogic Reflection
Actions Based on Reflection	

## Data Availability

The original contributions presented in the study are included in the article, further inquiries can be directed to the first author.

## Note

1 For brevity, unless otherwise specified, “students” refers to elementary and secondary school students, and “teachers” refers to elementary and secondary school teachers.

## Abbreviations

The following abbreviation is used in this manuscript:

MTSS	Multi-Tiered Systems of Support

## Appendix A

### Appendix A.1. Interview Protocol for Reflective Practitioners

Dear Teacher,

Thank you for participating in the interview on Homeroom Teachers’ Reflective Practice. The interview was designed to explore your prior experiences related to reflective practice. To enhance a meaningful and productive discussion, we kindly ask you to read the following questions in advance and recall your relevant experiences and feelings. We sincerely appreciate your time and input in this study.

**Interview Questions:**

1. Based on your registration form, you habitually reflect on your homeroom work. Could you briefly describe your reflective experiences and the typical approaches you used for reflection?
2. How did you develop this habit of reflection? Were there any significant figures or events that influenced you?
3. What aspects of your homeroom work did you usually reflect on? Could you describe in detail a particularly memorable reflective event?
4. Looking back on your reflective journey, could you identify distinct stages in its development? What rationale did you use to divide these stages?
5. What have you gained from engaging in reflective practice as a homeroom teacher?
6. Did you encounter any challenges when engaging in reflective practice? How did you cope with them? What kind of support do you think would have helped overcome them?
7. In your experience, what factors facilitated or hindered your engagement in reflective practice?

### Appendix A.2. Interview Protocol for Non-Reflective Practitioners

Dear Teacher,

Thank you for participating in the interview on Homeroom Teachers’ Reflective Practice. The interview was designed to explore your prior experiences related to reflective practice. To enhance a meaningful and productive discussion, we kindly ask you to read the following questions in advance and recall your relevant experiences and feelings. We sincerely appreciate your time and input in this study.

**Interview Questions:**

1. Based on your registration form, you did not habitually reflect on your homeroom work. In your course of being a homeroom teacher, did you encounter any confusion or difficult situations?
2. How did you typically deal with these confusions or difficult situations?
3. What is your perspective on the role of reflection in homeroom work?
